# Phosphorylation of VAMP3 couples IL-6 exocytosis to dendritic cell activation

**DOI:** 10.1242/jcs.264139

**Published:** 2025-10-14

**Authors:** Tongxiang Chen, Anthi Psoma, Shweta Mahajan, Martin ter Beest, Peter Linders, Giulia Franciosa, Frans Bianchi, Geert van den Bogaart, Harry Warner

**Affiliations:** ^1^ Department of Molecular Immunology, Groningen Biomolecular Sciences and Biotechnology Institute, University of Groningen, Groningen 9747 AG, the Netherlands; ^2^ Division of Immunobiology, Center for Inflammation and Tolerance, Cincinnati Children's Hospital, Cincinnati, OH 45229, USA; ^3^ Department of Medical BioSciences, Radboud University Medical Center, Nijmegen 6525 GA, the Netherlands; ^4^ Novo Nordisk Foundation Center for Protein Research, Faculty of Health and Medical Sciences, Copenhagen University, Copenhagen DK-2200, Denmark

**Keywords:** IL-6, VAMP3, Dendritic cell, Secretion, TIRF

## Abstract

Adaptive immunity is crucial for combating pathogens and generating immunological memory. Central to adaptive immunity are myeloid cells, which activate upon pathogen detection. Activation is essential for inflammatory cytokine release and requires a complex series of molecular events to facilitate cytokine expression. However, although the transcriptional machinery regulating cytokine expression is well defined, it is apparent that trafficking machinery also has to be reprogrammed to facilitate cytokine secretion. We demonstrate through quantitative total internal resonance fluorescence (TIRF) microscopy that short-term inflammatory stimulation with lipopolysaccharide (LPS) is sufficient to upregulate interleukin-6 (IL-6) secretion in dendritic cells. Through bioinformatic analysis of phosphoproteomic data, we demonstrate that the activation of dendritic cells rapidly reprograms SNARE-associated trafficking machinery. We link the enhanced rate of IL-6 secretion to phosphorylation of the SNARE protein VAMP3. This releases VAMP3 from the chaperone WDFY2, enabling the trafficking of IL-6-positive VAMP3-positive vesicles to the plasma membrane. VAMP3 then complexes with STX4, facilitating IL-6 secretion. Finally, we found that VAMP3-dependent IL-6 secretion is polarised, reconciling findings that, in dendritic cells, IL-6-positive vesicles are non-polarised, whereas VAMP3-positive vesicles are largely polarised.

## Teaser

**Highlighted Article:** Dendritic cells regulate IL-6 secretion upon LPS stimulation by disrupting interactions between the SNARE VAMP3 and its chaperone WDFY2, enabling VAMP3-positive vesicles to traffic to the plasma membrane and secrete IL-6.

## INTRODUCTION

Myeloid cell activation requires a complex series of molecular events to respond to pathogenic invasion and generate an adaptive immune response (Patente et al., 2019). Activated myeloid cells release cytokines to surrounding tissue at immunological synapses. Whilst much is known about the genetic regulation of cytokine production (Holloway et al., 2002), less is known about the trafficking machinery that facilitates cytokine secretion, and it has long been thought that cytokines utilise pre-established membrane trafficking circuits (Revelo et al., 2019).

Soluble *N*-ethylmaleimide-sensitive factor attachment protein receptor (SNARE) proteins are key regulators of membrane traffic that confer both identity to membranes and drive membrane fusion (Jahn and Scheller, 2006; Hong, 2005; Koike and Jahn, 2022). Both functions are achieved through the extreme specificity of each Q-SNARE for a limited set of cognate R-SNAREs. Whilst these functions of SNAREs have long been appreciated, it is becoming apparent that SNARE function can be rapidly modified through post-translational modifications (Warner et al., 2022; Malmersjö et al., 2016; Nair-Gupta et al., 2014).

We have previously shown that trafficking machinery plays an active role in controlling interleukin-6 (IL-6) synthesis: a pool of IL-6 at the perinuclear recycling compartment (PNRC) signals via its receptor IL-6R to dampen IL-6 transcription (Verboogen et al., 2019). Thus, trafficking machinery features as part of a larger feedback loop to fine-tune extracellular cytokine concentrations. Furthermore, VAMP3, an R-SNARE localising to the PNRC, is essential for IL-6 secretion (Boddul et al., 2014; Manderson et al., 2007; Verboogen et al., 2019).

We have previously observed increased VAMP3–STX4 complexing at the plasma membrane in dendritic cells following lipopolysaccharide (LPS) stimulation (Verboogen et al., 2017). Concomitant with this, we have shown that the IL-6 exocytosis rate is increased upon 24 h LPS stimulation (Verboogen et al., 2018). However, the mechanism underlying increased VAMP3-mediated exocytosis is unknown.

Here, we used total internal resonance fluorescence (TIRF) microscopy to address this. We show that brief LPS stimulation (1–4 h) increases the rate of IL-6 secretion. By reanalysis of our recently reported phosphoproteomics data (Warner et al., 2024), we link this to the phosphorylation of VAMP3 at serine 44 (serine 48 of mouse VAMP3), which promotes complexing of VAMP3 with STX4 at the plasma membrane, ultimately driving IL-6 secretion. We show that VAMP3 phosphorylation drives this by blocking the interaction of VAMP3 with the inhibitory chaperone WDFY2, freeing VAMP3-positive vesicles to complex with STX4. Finally, we demonstrate that, although IL-6-positive vesicles are evenly distributed throughout dendritic cells, their secretion pattern is asymmetric and thus partially polarised.

## RESULTS AND DISCUSSION

### LPS stimulation primes trafficking machinery via VAMP3 phosphorylation

**Fig. 1.**
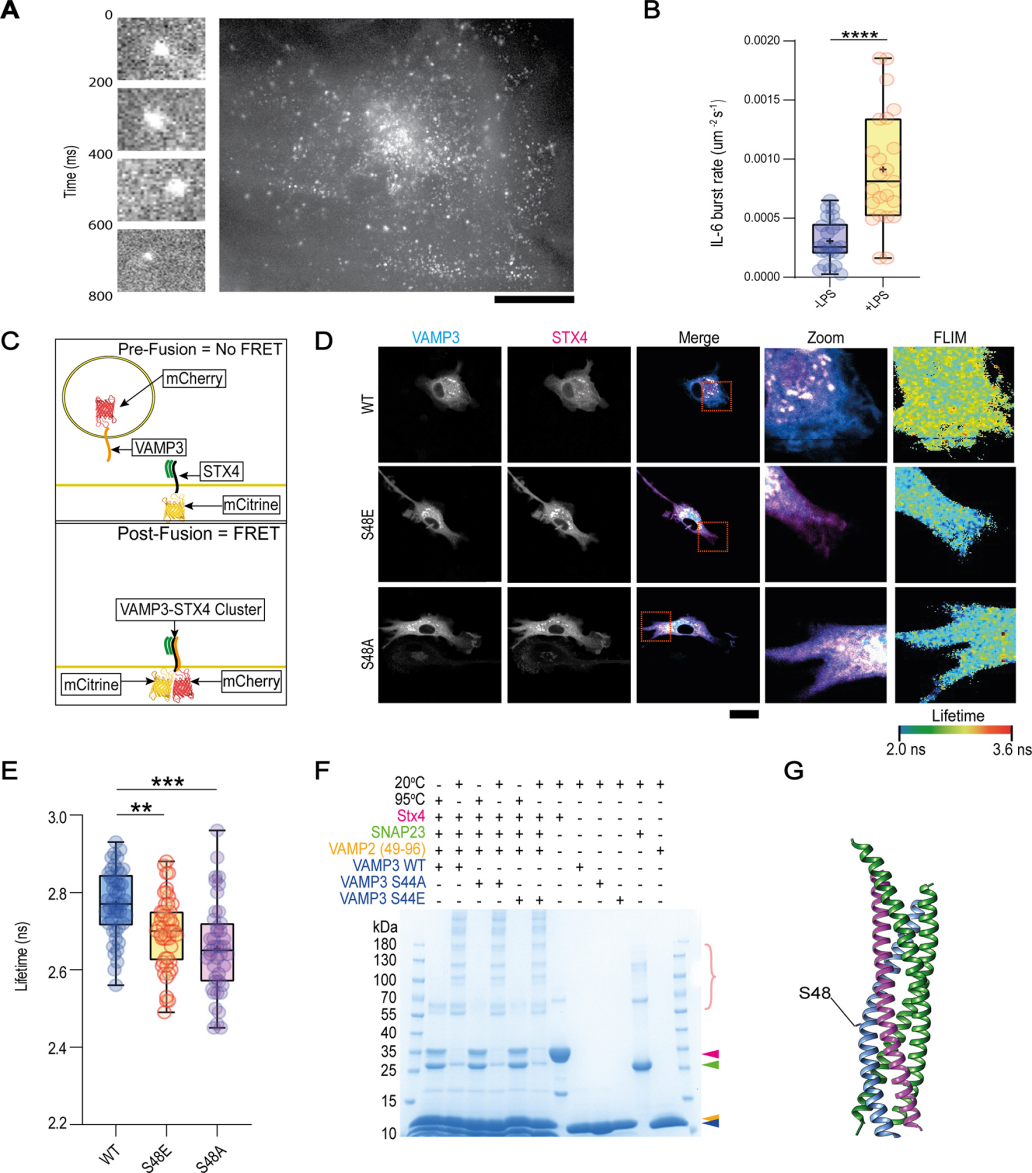
**LPS stimulation regulates IL-6 trafficking apparatus.** (A) Example TIRF imaging of IL-6 secretory events in dendritic cells expressing IL-6–GFP, with magnified views of GFP-burst events over a short time-period on the left. Scale bar: 20 μm. The main image is also shown in Fig. 3G. (B) Quantification of IL-6–GFP secretion rate in the absence or presence of short-term LPS stimulation (∼1–4 h prior to imaging). *n*=23 measurements per condition over three donors. Statistical significance calculated using a two-sided unpaired *t*-test (selected according to the distribution pattern of the data). ****P<0.0001. (C) Schematic of FRET–FLIM protocol that enables the monitoring of SNARE complexing. (D) Representative confocal micrographs and FLIM images of dendritic cells expressing STX4–mCitrine (magenta in merge image) and either WT, S48E or S48A variants of VAMP3–mCherry (cyan in merge image), as indicated. Dashed boxes indicate regions shown in zoom and FLIM images. Scale bar: 20 μm. (E) Quantification of STX4–mCitrine fluorescence lifetime in dendritic cells expressing different VAMP3 mutants indicates that both the phosphomimetic (S48E) and phosphodead (S48A) VAMP3 mutants complex more efficiently with STX4, compared to WT VAMP3. *n*≥43 measurements over four donors. One-way ANOVA with Tukey multiple comparison tests (test selected according to the distribution pattern of the data). **P<0.01; ***P<0.001. (F) *Ex cellulo* SNARE complex formation of purified cytosolic fragments of human VAMP3 WT, S44A and S44E (blue arrowhead) with a preformed acceptor complex of SNAP23 (green arrowhead), cytosolic fragment of STX4 (red arrowhead) and VAMP2(49–96) (orange arrowhead) analysed by SDS-PAGE and Coomassie Blue staining. Ternary SNARE complexes (multiple bands above ∼55 kDa; pink bracket) are SDS resistant at 20°C but disassemble at 95°C. Gel shown represents a single experiment. (G) Crystal structure of SNARE complex with rat VAMP3 (Lyubimov et al., 2016; PDB ID:5KJ7), with serine 48 indicated. Colours as in F. For box-and-whisker plots in B and E, box represents 25th to 75th percentile, whiskers represent maximum and minimum values, middle band represents the data median and+represents the data mean.

To study short-term changes to trafficking machinery following inflammatory stimulation, we overexpressed GFP-tagged IL-6 in human peripheral blood monocyte-derived dendritic cells and imaged secretory events in the absence or short-term presence of LPS using TIRF microscopy, as previously described for GFP–IL-6 (Verboogen et al., 2018). This revealed that short-term LPS stimulation (imaging between 1 h and 4 h) of dendritic cells was sufficient to upregulate the rate of IL-6 secretion (Fig. 1A,B; Movies 1, 2). This time frame was selected as LPS-induced IL-6 accumulates in the PNRC prior to secretion, with accumulation peaking at 4 h of stimulation (Verboogen et al., 2019). These findings are in line with our previous observations that overnight LPS stimulation increases the exocytosis rate of IL-6-carrying vesicles (Verboogen et al., 2018) and show that this increase already occurs within 1–4 h after pathogenic stimulation.

As short-term LPS stimulation drives IL-6 secretion, we examined our previously published human dendritic cell phosphoproteomic data (Warner et al., 2024) to identify novel phosphorylation and dephosphorylation events that regulate SNAREs and SNARE-associated proteins, using STRING software (Szklarczyk et al., 2019) with a score cut-off value of 0.7. A STRING score above this cut-off value indicates, based on a variety of metrics (including biochemical data and co-expression data, among others), that there is at least 70% chance of an interaction (direct or indirect) being biologically meaningful. This analysis revealed a network of proteins that interact with VAMP3 (including VAMP3 itself) and are rapidly phosphorylated in response to LPS stimulation (Fig. S1A; data from Warner et al., 2024). Further inspection of the Warner et al. (2024) dataset revealed two VAMP3 phosphorylation events. One at serine 44 (significant at 1 h and 4 h LPS stimulation; Fig. S1B) and the other at serine 11 (significant at 4 h LPS stimulation; Fig. S1C). Furthermore, we could not detect a change in VAMP3 protein levels in our total cell proteomics data (again from Warner et al., 2024) (Fig. S1D). We therefore chose to follow up the serine 44 phosphorylation event as, not only is this event more rapid, having been observed at 1 h and 4 h post-stimulation (Fig. S1E; Warner et al., 2024), the site is highly conserved among species (Fig. S1F). Furthermore, we previously observed that LPS induces increased complexing between VAMP3 and STX4 at the plasma membrane in dendritic cells (Verboogen et al., 2017), and STX4 is also highly conserved from mouse to human (Fig. S2).

### VAMP3 phosphorylation drives SNARE complex formation

As VAMP3 is consistently phosphorylated at serine 44 in response to LPS stimulation, we predicted that VAMP3 phosphorylation drives complexing with STX4, as VAMP3 complexes with STX4 in LPS-stimulated dendritic cells (Verboogen et al., 2017). To test this, we utilised Förster resonance energy transfer fluorescence lifetime imaging microscopy (FRET–FLIM). As we have previously described (Linders et al., 2021; Verboogen et al., 2017), this technique can be used to visualise SNARE complexes in living cells with organellar resolution. We used FRET–FLIM to determine whether this phosphorylation would alter the fusogenic activity of VAMP3 with STX4 (Fig. 1C). For these experiments, we overexpressed mouse variants of VAMP3 and STX4, which we believe is warranted given the high sequence similarity between the human and mouse proteins (Figs S1F and S2).

Analysing the lifetime of STX4–mCitrine in dendritic cells overexpressing mCherry-tagged wild-type (WT) VAMP3 (VAMP3–mCherry WT) or VAMP3 with point mutations at serine 48 (the mouse equivalent of human VAMP3 serine 44) revealed that both phosphomimetic (VAMP3–mCherry S48E) and phosphodead (VAMP3–mCherry S48A) variants are more effective at complexing with STX4 at the plasma membrane, relative to WT VAMP3 (Fig. 1D,E). Thus, the phosphorylation of VAMP3 is not driving SNARE complexing per se. Rather it seems that disruption of the OH group on the serine blocks an interaction between VAMP3 and an inhibitory chaperone, promoting VAMP3-positive vesicle trafficking and enabling VAMP3–STX4 complexing at the plasma membrane. Consistent with this, recombinantly purified cytosolic fragments of human VAMP3 (WT, S44E variant and S44A variant) showed equal potential for complexing *in vitro* with a preformed acceptor complex of SNAP23 and the cytosolic fragment of STX4 (Fig. 1F). Here, we took advantage of the fact that SNARE complex formation is greatly accelerated when a 1:1 acceptor complex of STX4 and SNAP23 is stabilised with the C-terminal half of the SNARE motif of VAMP2 (residues 49–96) (Pobbati et al., 2006). Moreover, in the crystal structure of the SNARE complex of rat VAMP3 with SNAP25 and STX1A (Lyubimov et al., 2016), serine 48 of rat VAMP3 (homologous to serine 44 of human VAMP3) points away from the SNARE bundle, supporting that this residue does not directly affect SNARE complex formation (Fig. 1G).

### VAMP3 phosphorylation results in dissociation of inhibitory chaperone WDFY2

**Fig. 2.**
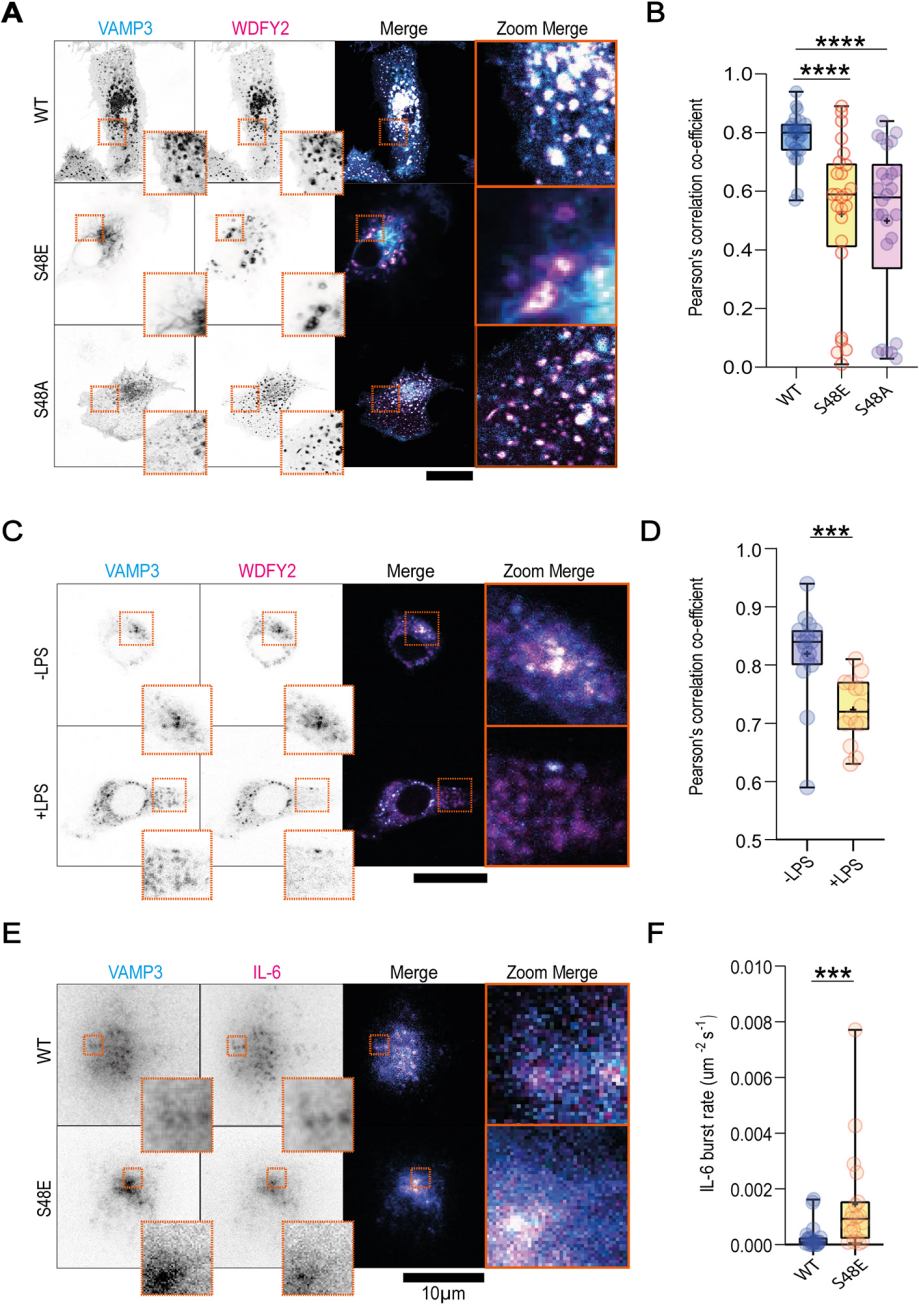
**WDFY2 releases VAMP3 in response to LPS stimulation.** (A) Example confocal micrographs of dendritic cells overexpressing GFP–WDFY2 (magenta in merge image) and VAMP3–mCherry variants (cyan in merge image), as indicated. (B) Pearson's correlation coefficient of GFP–WDFY2 with VAMP3–mCherry WT, phosphomimetic (S48E) or phosphodead (S48A) variants reveals that the interaction of VAMP3 with WDFY2 is dependent on the OH group of serine 48. *n*≥25 measurements per condition over three donors. (C) Example confocal micrographs of dendritic cells overexpressing GFP–WDFY2 (magenta in merge image) and WT VAMP3–mCherry (cyan in merge image) in the absence or presence of LPS. (D) Pearson's correlation coefficient of GFP–WDFY2 with WT VAMP3–mCherry in the absence or presence of LPS reveals that the interaction of VAMP3 with WDFY2 is disrupted in response to LPS stimulation. *n*≥14 measurements over three donors. (E) Two-color TIRF microscopy showing overlap of IL-6–GFP (magenta in merge image) with VAMP3–mCherry trafficking vesicles for WT and S48E variants of VAMP3–mCherry (cyan in merge image). (F) Quantification of IL-6–GFP bursts reveals that overexpression of S48E VAMP3–mCherry is sufficient to drive IL-6–GFP secretion. Results are for 19 measurements from three donors. In A, C and E, dashed boxes indicate regions shown as magnified insets. Scale bars: 20 μm (unless otherwise stated). For box-and-whisker plots, box represents 25th to 75th percentile, whiskers represent maximum and minimum values, middle band represents the data median and+represents the data mean. One-way ANOVA with Tukey multiple comparison test was used in B; two-tailed unpaired *t*-tests were used in D and F (selected according to the distribution pattern of the data). ***P<0.001; ****P<0.0001.

WDFY2 has previously been shown to restrain both VAMP2 and VAMP3 vesicle dynamics, through binding to VAMP3 (Fritzius et al., 2007; Sneeggen et al., 2019). To test whether the VAMP3 serine 44/serine 48 OH group is crucial for WDFY2 binding, we co-expressed WDFY2 with either WT VAMP3, VAMP3 S48E or VAMP3 S48A in dendritic cells, and imaged them using confocal microscopy (Fig. 2A). Pearson correlation analysis, which is insensitive to absolute expression levels, revealed a significant decrease in colocalisation of VAMP3 S48E or VAMP3 S48A with WDFY2 compared to WT VAMP3 (Fig. 2B). Furthermore, immunoprecipitated GFP–WDFY2, overexpressed in HEK293 cells, could only co-immunoprecipitate WT VAMP3–mCherry, but failed to co-immunoprecipitate S48E or S48A VAMP3–mCherry (Fig. S3A).

We then confirmed that LPS stimulation of dendritic cells reduces WT VAMP3–WDFY2 colocalisation in overexpressing cells (Fig. 2C,D). We also verified that *WDFY2* is endogenously expressed in monocyte-derived dendritic cells using analysis data from Lee et al. (2014). We found that *WDFY2* is expressed and undergoes a small but significant suppression following LPS stimulation (Fig. S3B). We then verified that VAMP3 S48E overexpression is sufficient to upregulate the rate of IL-6 secretion, relative to VAMP3 WT (Fig. 2E,F; Movies 3, 4).

### VAMP3 secretion is asymmetric in dendritic cells

**Fig. 3.**
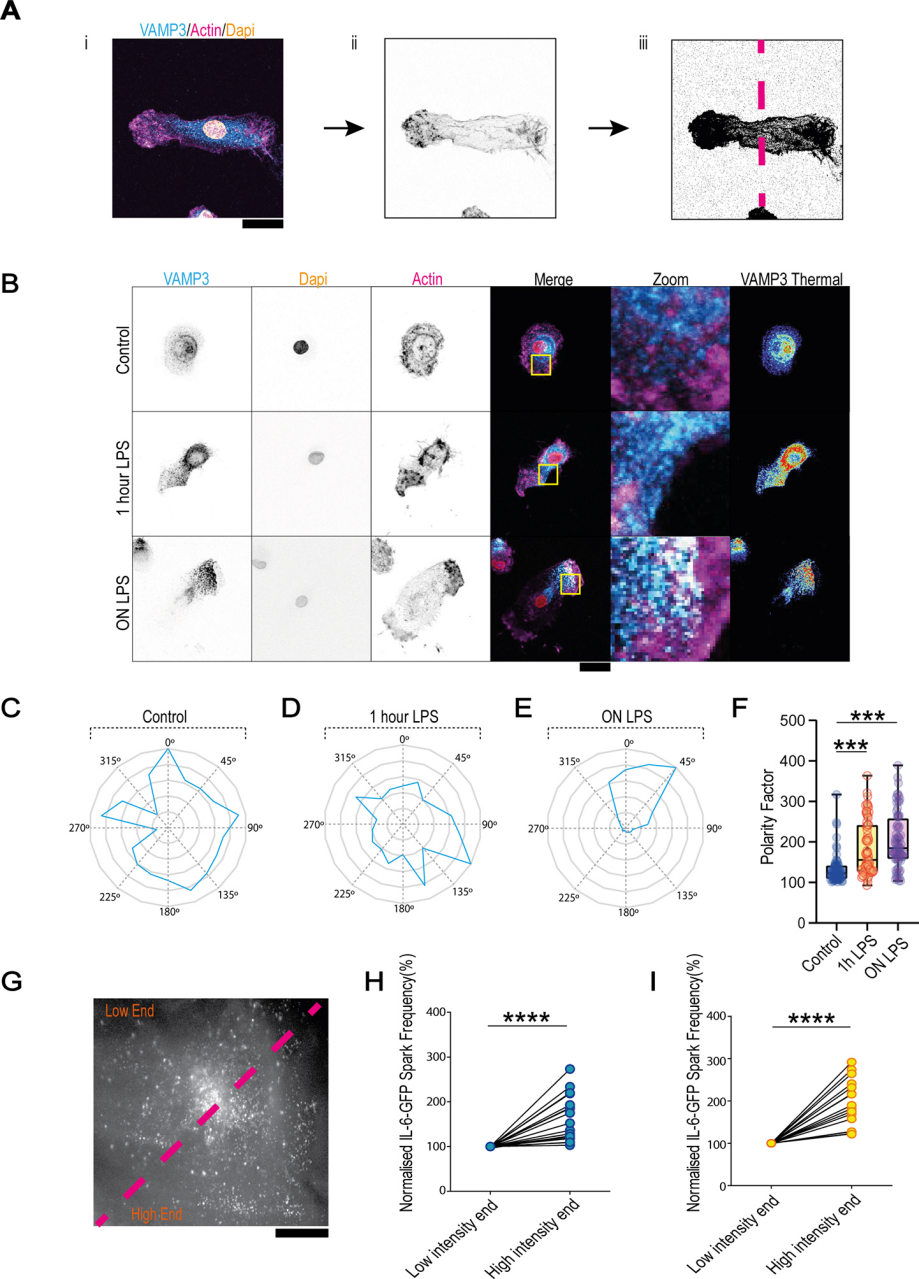
**IL-6 secretion is polarised.** (A) Schematic for determining VAMP3 polarity. The cell (i) is outlined using a fluorescence intensity threshold generated from phalloidin staining (ii). The cell is then segmented perpendicular to the longest axis into two areas of equal projected size (iii, dashed line) so that VAMP3 relative intensity can be measured in each area, and the polarity factor calculated by dividing the highest intensity by the lowest intensity, then multiplying by 100. (B) Example confocal micrographs of dendritic cells stained for VAMP3 (cyan in merge image), DNA (DAPI, green in merge image) and F-actin (magenta in merge image), along with a heat map of VAMP3 signal intensity (thermal look-up table). Cells were either unstimulated or were stimulated with LPS for 1 h or overnight (ON). Box indicates region shown in zoom image. (C) Example radar plot of normalised VAMP3 distribution throughout an unstimulated dendritic cell. (D) Example radar plot of normalised VAMP3 distribution throughout a dendritic cell stimulated for 1 h with LPS. (E) Example radar plot of normalised VAMP3 distribution throughout a dendritic cell stimulated overnight with LPS. (F) VAMP3 polarisation increases after 1 h and overnight LPS stimulation. *n*≥67 measurements per condition over three donors. Box represents 25th to 75th percentile, whiskers represent maximum and minimum values, middle band represents the data median and+represents the data mean. (G) Example TIRF image from Fig. 1A, segmented (dashed line) to determine whether IL-6 secretion is polarised. (H) Quantification of IL-6–GFP secretion rate at ‘high’ and ‘low’ ends of the cell in inactive dendritic cells. *n*=19 measurements over three donors. (I) Quantification of IL-6–GFP secretion rate at ‘high’ and ‘low’ ends of the cell in LPS-activated dendritic cells. *n*=17 measurements over three donors. Scale bars: 20 μm. One-way ANOVA with Tukey multiple comparison test in F; two-sided paired *t*-test in H and I (with test selected according to the distribution pattern of the data). ***P<0.001; ****P<0.0001.

As VAMP3 phosphorylation releases VAMP3-positive vesicles from WDFY2, we examined the impact of LPS stimulation on endogenous VAMP3-positive vesicle distribution. To do this we segmented the cell using phalloidin staining, bisected it perpendicular to the longest axis into two halves with equal projected surface areas (Fig. 3A) and calculated the polarity index of VAMP3. Here, the polarity index was defined as the total high-end fluorescence intensity divided by the low-end fluorescence intensity, multiplied by 100. Consistent with our hypothesis, and with previous literature (Santalla Méndez et al., 2023; Veale et al., 2011), stimulation with LPS drove the spatial polarisation of VAMP3-positive vesicles (Fig. 3B–F). We also verified that WDFY2 is likewise polarised (Fig. S3C,D). Furthermore, we also observed trafficking of VAMP3-positive vesicles towards F-actin-enriched regions (Fig. S3E,F), suggesting that polarised IL-6 secretion is coupled to F-actin protrusion formation. However, this is inconsistent with previous work showing that IL-6 secretion, unlike TNFα secretion, is unpolarised, despite being dependent on VAMP3 (Bajno et al., 2000; Fields et al., 2007; Verboogen et al., 2019; Murray et al., 2005). We therefore checked the distribution of vesicles carrying IL-6–GFP (in the absence or presence of LPS), using TIRF microscopy (Fig. S4A–C). This revealed that the distribution of intracellular IL-6–GFP is indeed unpolarised. Based on these data, we predicted that although the IL-6 vesicle distribution might be unpolarised, the secretory events (which are VAMP3 dependent) are polarised.

To test our hypothesis, we analysed the distribution of IL-6–GFP secretory events as observed by TIRF microscopy (Fig. 1A,B). To quantify this, we again segmented the imaged cell area into two areas with the same projected size (Fig. 3G). The polarity index of IL-6–GFP burst distribution revealed that secretory events were not uniformly distributed over the imaged cell area but instead were more localised to one side of the cell (Fig. 3H,I). This revealed that IL-6 secretion is indeed asymmetric and thus polarised (Fig. 3G–I).

**Fig. 4.**
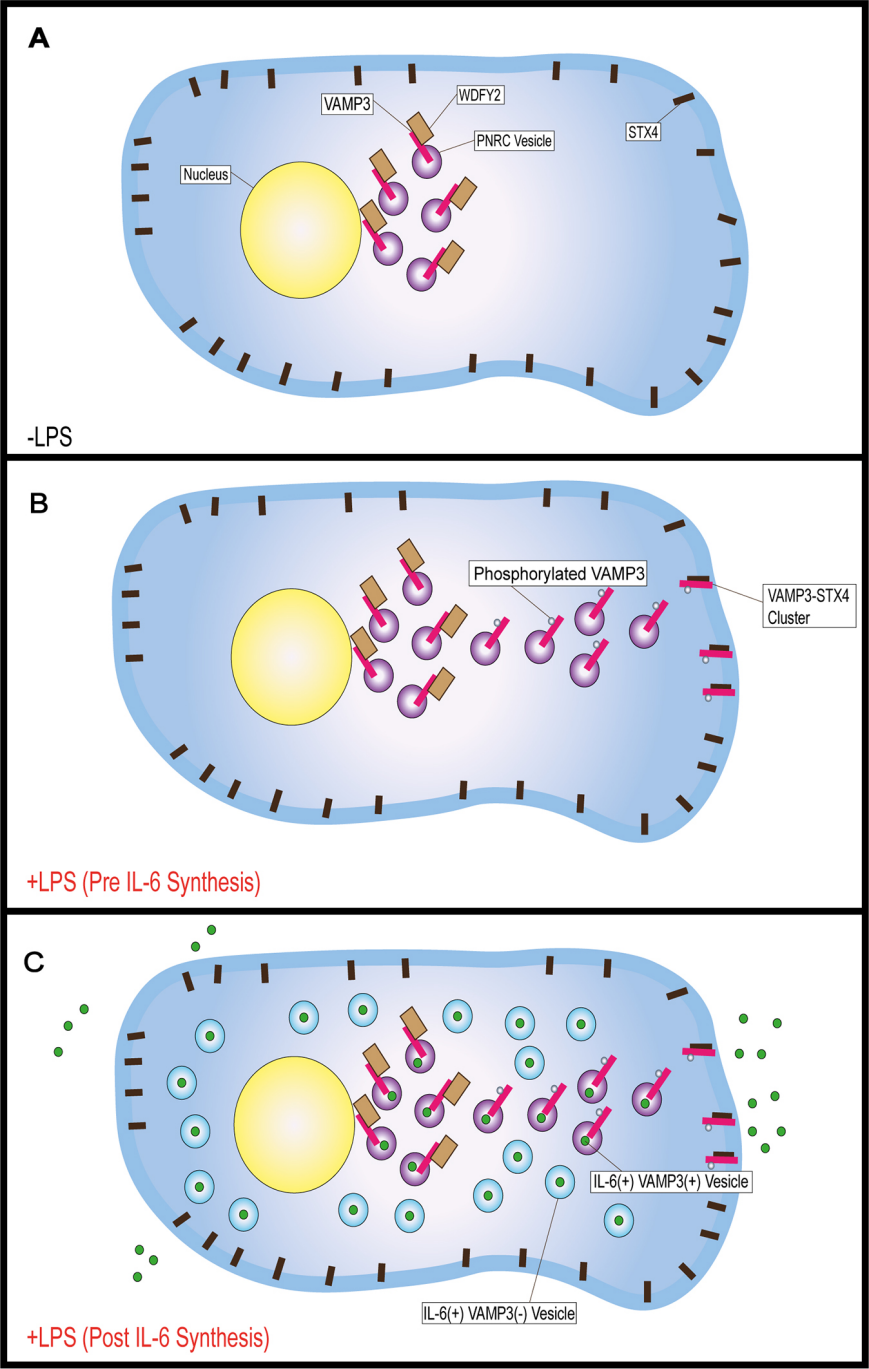
**Model schematic.** (A) Inactive dendritic cells restrict VAMP3-positive (PNRC) vesicle movement and/or complexing with STX4 through the formation of a WDFY2–VAMP3 complex. (B) LPS stimulation primes the cell for IL-6 secretion, via the phosphorylation of VAMP3 at serine 44, disrupting the VAMP3–WDFY2 complex. VAMP3-positive vesicles then move to the plasma membrane, enabling VAMP3 to complex with STX4. (C) Newly synthesised IL-6 is trafficked through the biosynthetic trafficking pathway to the PNRC, through which it is secreted in a VAMP3-dependent manner. The appearance of IL-6-positive VAMP3-negative vesicles obscures the polarised delivery of IL-6-positive VAMP3-positive vesicles to the plasma membrane.

In summary, our results suggest that in inactive dendritic cells, VAMP3 forms a complex with WDFY2 on VAMP3-positive endosomes, restricting the traffic of the associated cargo (Fig. 4A). LPS-induced activation leads to the VAMP3 phosphorylation priming the secretory machinery prior to IL-6 synthesis (Fig. 4B). This destabilises the interaction of VAMP3 with WDFY2, enabling VAMP3 to complex with STX4 at the plasma membrane. Once synthesised, IL-6-carrying vesicles are evenly distributed throughout the cell. IL-6 is eventually shuttled to VAMP3-containing recycling endosomal vesicles, which facilitates the polarised secretion of IL-6 (Fig. 4C).

## MATERIALS AND METHODS

### Plasmids

For mammalian expression, pmCitrine-N1-STX4 (Addgene #92422) and pmCherry-N1-VAMP3 (Addgene #92423) were generated for a previous study (Verboogen et al., 2017). pmCherry-N1-VAMP3 S48E, pmCherry-N1-VAMP3 S48A and pEGFP-N1-WDFY2 (human) were generated as synthetic constructs (Genscript) and have been deposited at Addgene.

The SNAP23 and VAMP2 (49–96) constructs for bacterial expression have been described previously (Antonin et al., 2002; Pobbati et al., 2006). Human VAMP3 WT, S44A and S44A (lacking the transmembrane domain, residues 1–77), and human STX4 (lacking the transmembrane domain; residues 1–271 with 272C) were generated as synthetic genes (Genscript) with codon optimisation for human expression and were cloned into the NdeI/XhoI site of pET28a (+) (Verboogen et al., 2019; Addgene #111933). Plasmids have been deposited at Addgene.

### Antibodies

The following primary antibodies were used in this study: rabbit polyclonal anti-VAMP3 (Abcam, Cambridge, UK; ab5789), rabbit polyclonal anti-WDFY2 (Abcam, ab237623), rabbit polyclonal anti-GFP antibody (Rockland, 600-401-15) and mouse monoclonal anti-GAPDH antibody (Santa Cruz, sc-365062). The following secondary stainings were used in this study: Alexa Fluor 488 Phalloidin (Invitrogen, A12379; 1:200), goat anti-rabbit IgG Alexa Fluor 647 (Invitrogen, A27040; 1:200), horseradish peroxidase (HRP)-linked horse anti-mouse IgG antibody (Cell Signaling Technology, 7076) and HRP-linked goat anti-rabbit IgG antibody (Cell Signaling Technology, 7074).

### Western blotting

Samples were resolved on 4–20% Mini-Protein TGX precast gels (Bio-Rad) at 120V. The gels were then blotted to PVDF membranes at 90V for 60 min. Blots were blocked in 5% skim milk powder (Fisher Scientific) in TBS containing 0.01% Tween 20 (TBST) at room temperature for 30 min to 1 h. After blocking, blots were washed three times in TBST and then probed with rabbit polyclonal anti-WDFY2 antibody (Abcam, ab237623) at 1:500, rabbit polyclonal anti-cellubrevin (VAMP3) antibody at 1:1000 (Abcam, ab5789), rabbit polyclonal anti-GFP antibody (Rockland, 600-401-15) at 1:1000 or mouse monoclonal anti-GAPDH antibody at 1:1000 (Santa Cruz, sc-365062) at 4°C overnight. The next day, blots were washed three times in TBST and then labelled with HRP-linked anti-mouse IgG antibody (Cell Signaling Technology, 7076) or HRP-linked anti-rabbit IgG antibody (Cell Signaling Technology, 7074), both at 1:5000, at room temperature for 2 h. After washing three times, the blots were scanned using the Odyssey CLx Infrared Imaging System and analysed using ImageStudio (LI-COR). See western blot transparency Fig. S5 for total blots.

### Cells

Monocyte-derived dendritic cells were obtained by differentiating human peripheral blood CD14-positive monocytes with interleukin-4 (300 µg/ml; Miltenyi, 130-093-924) and granulocyte-macrophage colony-stimulating factor (GM-CSF; 450 µg/ml; Miltenyi ,130-093-867) for 6 days in RPMI (Thermo Fisher Scientific, 11530586) supplemented with 10% serum (FBS; Thermo Fisher Scientific, 10309433), antibiotics (100 μg ml^−1^ penicillin, 100 µg ml^−1^ streptomycin and 0.25 µg ml^−1^ amphotericin B; Gibco, 15240062) and 2 mM glutamine (Gibco, 21875-034). Monocytes were isolated from the blood of healthy donors (informed consent and consent to publish obtained, approved by the ethical committee of the Dutch blood bank, Sanquin) as previously described (de Vries et al., 2002). LPS (O111:B4, Sigma Aldrich, 32160405) stimulation was carried out overnight unless otherwise stated. HEK293 cells (ATCC) were cultured as previously described (Ioannidis et al., 2025).

### Confocal microscopy

Cells were seeded onto glass coverslips for at least 12 h before fixation. Cells were fixed in 4% paraformaldehyde (PFA) for 15 min at room temperature. Cells fixed in PFA were permeabilised for 5 min in a 0.1% (v/v) Triton X-100 solution. Cells were blocked in a 20 mM glycine, 3% bovine serum albumin (BSA) PBS-based solution for 1 h before antibody staining.

Transient transfection of dendritic cells was achieved using the Neon transfection system (Thermo Fisher Scientific). 1.2 million cells were washed with PBS and suspended in 115 µl of buffer R with 5 µg of DNA. Cells were pulsed twice for 40 ms at 1000 V. Cells were then transferred to Phenol Red-free RPMI (Thermo Fisher Scientific, 32404-014/11564456) with 20% serum for at least 4 h before imaging. Live-cell imaging was performed in OptiKlear solution (Abcam, ab275928).

Images were collected with a Zeiss LSM 800 microscope equipped with a Plan-Apochromat (63×/1.4) oil DIC M27 (FWD=0.19 mm) objective (Zeiss). Images were acquired using the ZEN software (Zeiss, version 2.3). DAPI (Sigma-Aldrich, 32670) was excited by a 405 nm laser, and Alexa Fluor 488 Phalloidin was excited by a 488 nm laser. For *Z*-series, a slice interval of 0.31 µm was used. Airyscan microscopy was performed with a Zeiss LSM 800 Airyscan microscope. A *Z*-interval of 0.17 µm was used. Images were acquired using the ZEN software (version 2.3). Images were subject to Airyscan processing following acquisition.

### TIRF microscopy and analysis

TIRF was performed on an Olympus IX51 inverted microscope equipped with a 60×/1.49 oil immersion objective cell TIRF illuminator (Olympus). Excitation was done with a 488 nm laser. Fluorescence emission was collected with an appropriate dichroic mirror and Photometrics Prime Express sCMOS camera.

Images were recorded at 37°C as time-lapse movies of 1000 frames over an interval of 100 ms. The number of bursts/flashes was analysed from time-lapse movies of monocyte-derived dendritic cells expressing IL-6–GFP using the ImageJ plugin xySpark (Steele and Steele, 2014).

The data from xySpark (frame number, number of sparks, time) were imported into GraphPad Prism (v 8.4.3) to plot graphs and perform statistical analysis. We performed calibration to convert the cell surface area obtained in pixels using ImageJ, to obtain the cell area in micrometres. The results are represented as the number of sparks across different conditions and as bursts s^−1^ µm^−2^.

### FRET–FLIM

Live imaging was performed on a Leica SP8 SMD system at 37°C, equipped with an HC PL APO CS2 63×/1.20 water objective. Fluorophores were excited with a pulsed white-light laser, operating at 80 MHz. mCitrine was excited at 514 nm, two separate HyD detectors were used to collect photons, set at 521–565 nm and 613–668 nm, respectively. Photons were collected for 1 min, and lifetime histograms of the donor fluorophore were fitted with mono-exponential decay functions convoluted with the microscope instrument response function in Leica LAS X. Data analysis was performed with the FLIMfit software tool (version 5.1.1; Warren et al., 2013) developed at Imperial College, London. All data were fitted with exponential decay functions convoluted with an instrument response function (IRF). The lifetime of STX4–mCitrine was then analysed at the cell periphery (approximating the plasma membrane).

### *Ex cellulo* complex formation

SNAREs were expressed in BL21 (DE3) *E. coli*, induced at an optical density at 600 nm (OD_600_) of ∼0.5 with isopropyl β-D-1-thiogalactopyranoside (IPTG; I6758, Sigma) in a final concentration of 0.5 mM for 6 h. Cell pellets were incubated for 30 min at room temperature at 4 ml/g with extraction buffer: 20 mM HEPES pH 7.4, 500 mM NaCl, 8 mM imidazole, 1 mM tris-(2-carboxyethyl)phosphine hydrochloride (TCEP), 1 mM MgCl_2_, 1 mM phenylmethanesulfonyl fluoride (PMS), DNAse (2 μg/ml; Invitrogen, 18068-015) and lysozyme (0.2 mg/ml; L6876, Sigma). Then, the same volume of extraction buffer as used before with an additional 10% sodium cholate hydrate (3α,7α,12α-trihydroxy-5β-cholan-24-oic acid sodium salt) was added. After another incubation step of 15 min at room temperature, 4 M urea was added, and the reaction was further incubated for 30 min at room temperature. The cells were lysed via sonication (MSE, Soniprep 150 Plus) with ten cycles of 15 s sonication (amplitude, 6–8; 30 s off and on ice). After taking a sample, cell debris was removed via centrifugation (Thermo Scientific, Sorval Lynx 40001) at 27,000 ***g*** at 4°C for 30 min. After taking a sample from the cell-free extract (CFE), the chromatography column (BioRad, Poly-Prep Chromatography Column) was prepared by adding 1 ml of resin (His-select nickel affinity gel, P6611, Sigma), washing the column twice with 5 ml ice-cold MilliQ water and equilibrating it with 5 ml of wash buffer: 20 mM HEPES pH 7.4, 500 mM NaCl, 20 mM imidazole, 1 mM TCEP, 1% 3-[(3-cholamidopropyl) dimethylammonio]-1-propanesulfonate hydrate (CHAPS).

CFE was added to the column and mixed well with the resin. After incubating for 2 h on a roller at 4°C, the column was opened and a sample from the flow-through was collected. The column was washed five times with 500 μl of wash buffer and then five times with 500 μl elution buffer (20 mM HEPES pH 7.4, 500 mM NaCl, 400 mM imidazole, 1 mM TCEP, 1% CHAPS). Samples were collected and the concentration was measured by absorption at 280 nm. 50 μl of thrombin (5 mg/ml in 50% glycerol=1 U/ml; MP Biomedicals, 215416301) was added to the elution with the highest concentration, in order to cleave the His-tag from the SNARE protein. The reaction was performed in dialysis cups (Thermo Scientific, Slide-A-Lyzer MINI Dialysis Device, 2 K MWCO, 0.1 ml) with dialysis buffer (20 mM HEPES pH 7.4, 100 mM NaCl, 0.2 mM TCEP and 1% CHAPS) overnight at 4°C in a beaker while stirring.

For further purification of VAMP3 mutants, cation exchange chromatography (BioRad, NGC QuestTM 10 Chromatography System #7880001) was performed. As a column, the SOURCE 15S 4.6/100 PE (cation) (Cytiva) was used. Prior to loading, the column was equilibrated with AKTA-A buffer (20 mM HEPES pH 7.4, 1 M NaCl, 0.2 mM TCEP and 1% CHAPS). For elution, a gradient volume of 15 column volumes to a sodium chloride concentration of 0.5 M AKTA-B buffer (AKTA-A with 1 M NaCl) was used. This segment was followed by stepping up the gradient to a salt concentration of 1 M (100% AKTA-B) and then holding it at 1 M for 3 ml before re-equilibration the column with 3 ml of AKTA-A. Before storing the fractions at −20°C, glycerol was added in a final concentration of 10% as a lyoprotectant.

For further purification of the STX4 complex with SNAP23 and VAMP2 (49–96), 100 mM of each protein was mixed. The mixture was incubated overnight at room temperature. The complex was then purified using size exclusion chromatography (BioRad, NGC QuestTM 10 Chromatography System #7880001). Prior to loading, the column (ENrich SEC 70 10×300 mm column) was equilibrated with dialysis buffer (20 mM HEPES pH 7.4, 100 mM NaCl, 0.2 mM TCEP and 1% CHAPS). The protein concentration of the elution fractions was determined by BCA Assay (Pierce BCA Protein Assay Kit).

For SNARE complex formation, 10 mM of the STX4 complex with SNAP23 and VAMP2 (49–96) was mixed with VAMP3 and incubated at room temperature overnight. Further analysis was done by SDS-PAGE and Coomassie Blue staining. Samples were mixed with 2× Laemmli buffer and heated for 10 min at 95°C or not heated. BioRad precast protein gels (4–20% Mini-PROTEAN TGX Precast Protein Gels, 15-well, 15L #4561096) were used for the SDS-PAGE.

### Immunoprecipitation

HEK293 cells were seeded in 75 mm culture dishes and transfected at ∼70% confluency using jetPEI (Polyplus), according to the manufacturer's instructions. Cells were co-transfected with pEGFP-N1-WDFY2 and one of the following constructs: pmCherry-N1-VAMP3, pmCherry-N1-VAMP3 S48A, pmCherry-N1-VAMP3 S48E. After 48 h of transfection, cells were harvested for co-immunoprecipitation experiments.

Transfected HEK293 cells were lysed in ice-cold RIPA buffer (Thermo Fisher Scientific, 89901) containing protease inhibitor cocktail (Roche, 04693116001). The lysates were incubated on ice for 30 min and then centrifuged at 15,000 ***g*** for 15 min at 4°C to remove cellular debris. The supernatants were collected, and total protein concentrations were determined using the Pierce BCA Protein Assay Kit (Thermo Fisher Scientific, 23225) according to the manufacturer's instructions.

Equal amounts of total protein (2 mg) were used for each immunoprecipitation. The lysates were then incubated overnight at 4°C with 4.8 μg of anti-GFP antibody (Rockland, 600-401-215) under constant rotation. The following day, 0.125 mg of protein A/G magnetic beads (Thermo Fisher Scientific, 88802) were added and incubated for an additional 1 h at room temperature to capture immune complexes.

Beads were collected using a magnetic stand and washed three times with wash buffer (TBST) to remove non-specifically bound proteins. Bound complexes were eluted by boiling the beads in 2× SDS sample buffer (1610747, Bio-Rad) for 10 min at 95°C. Proteins were resolved by SDS-PAGE and transferred to PVDF membranes (Millipore). Co-immunoprecipitated proteins were detected by western blotting using specific antibodies against WDFY2 and VAMP3 proteins, separately.

## Supplementary Material

10.1242/joces.264139_sup1 Supplementary information

## References

[JCS264139C1] Antonin, W., Dulubova, I., Arac, D., Pabst, S., Plitzner, J., Rizo, J. and Jahn, R. (2002). The N-terminal domains of syntaxin 7 and vti1b form three-helix bundles that differ in their ability to regulate SNARE complex assembly. *J. Biol. Chem.* 277, 36449-36456. 10.1074/jbc.M204369200 12114520

[JCS264139C2] Bajno, L., Peng, X.-R., Schreiber, A. D., Moore, H.-P., Trimble, W. S. and Grinstein, S. (2000). Focal Exocytosis of Vamp3-containing vesicles at sites of phagosome formation. *J. Cell Biol.* 149, 697. 10.1083/jcb.149.3.697 10791982 PMC2174839

[JCS264139C3] Boddul, S. V., Meng, J., Dolly, J. O. and Wang, J. (2014). SNAP-23 and VAMP-3 contribute to the release of IL-6 and TNFα from a human synovial sarcoma cell line. *FEBS J.* 281, 750-765. 10.1111/febs.12620 24373201

[JCS264139C5] de Vries, I. J. M., Eggert, A. A. O., Scharenborg, N. M., Vissers, J. L. M., Lesterhuis, W. J., Boerman, O. C., Punt, C. J. A., Adema, G. J. and Figdor, C. G. (2002). Phenotypical and functional characterization of clinical grade dendritic cells. *J. Immunother.* 25, 429-438. 10.1097/00002371-200209000-00007 12218781

[JCS264139C6] Fields, I. C., Shteyn, E., Pypaert, M., Proux-Gillardeaux, V., Kang, R. S., Galli, T. and Fölsch, H. (2007). v-SNARE cellubrevin is required for basolateral sorting of AP-1B–dependent cargo in polarized epithelial cells. *J. Cell Biol.* 177, 477. 10.1083/jcb.200610047 17485489 PMC2034334

[JCS264139C7] Fritzius, T., Frey, A. D., Schweneker, M., Mayer, D. and Moelling, K. (2007). WD-repeat-propeller-FYVE protein, ProF, binds VAMP2 and protein kinase Czeta. *FEBS J.* 274, 1552-1566. 10.1111/j.1742-4658.2007.05702.x 17313651

[JCS264139C8] Holloway, A. F., Rao, S. and Shannon, M. F. (2002). Regulation of cytokine gene transcription in the immune system. *Mol. Immunol.* 38, 567-580. 10.1016/S0161-5890(01)00094-3 11792425

[JCS264139C9] Hong, W. (2005). SNAREs and traffic. *Biochim. Biophy. Acta –Mol. Cell Res.* 1744, 120-144. 10.1016/j.bbamcr.2005.03.014 15893389

[JCS264139C10] Ioannidis, M., van Dijk, H., Muntjewerff, E. M., Chirasani, V. R., Warner, H., van den Dool, W., Grijpstra, P., Bianchi, F., Mahata, S. K. and van den Bogaart, G. (2025). Inflammation promotes proteolytic processing of the prohormone chromogranin a by macrophages. *J. Endocr. Soc.* 9, bvaf090. 10.1210/jendso/bvaf090 40458085 PMC12127132

[JCS264139C11] Jahn, R. and Scheller, R. H. (2006). SNAREs — engines for membrane fusion. *Nat. Rev. Mol. Cell Biol.* 7, 631-643. 10.1038/nrm2002 16912714

[JCS264139C12] Koike, S. and Jahn, R. (2022). SNARE proteins: zip codes in vesicle targeting? *Biochem. J.* 479, 273-288. 10.1042/BCJ20210719 35119456 PMC8883487

[JCS264139C13] Lee, M. N., Ye, C., Villani, A.-C., Raj, T., Li, W., Eisenhaure, T. M., Imboywa, S. H., Chipendo, P. I., Ran, F. A., Slowikowski, K. et al. (2014). Common genetic variants modulate pathogen-sensing responses in human dendritic cells. *Science* 343, 1246980. 10.1126/science.1246980 24604203 PMC4124741

[JCS264139C14] Linders, P. T. A., Howell, S. E. L., Brady, M., Xu, X. and McNeil, K. (2021). Congenital disorder of glycosylation caused by starting site-specific variant in syntaxin-5. *Nat. Commun.* 12, 1-15. 10.1038/s41467-020-20314-w 34711829 PMC8553859

[JCS264139C15] Lyubimov, A. Y., Di Palma, S., Diao, J., Lai, Y., Pfuetzner, R. A., Wang, A. L., McMahon, M. A., Hayer, A., Porteus, M., Bodenmiller, B. et al. (2016). Advances in X-ray free electron laser (XFEL) diffraction data processing applied to the crystal structure of the synaptotagmin-1/SNARE complex. *eLife* 5, e18740. 10.7554/ELIFE.18740 27731796 PMC5094853

[JCS264139C16] Malmersjö, S., Di Palma, S., Diao, J., Lai, Y., Pfuetzner, R. A., Wang, A. L., McMahon, M. A., Hayer, A., Porteus, M., Bodenmiller, B. et al. (2016). Phosphorylation of residues inside the SNARE complex suppresses secretory vesicle fusion. *EMBO J.* 35, 1810. 10.15252/embj.201694071 27402227 PMC5010044

[JCS264139C17] Manderson, A. P., Kay, J. G., Hammond, L. A., Brown, D. L. and Stow, J. L. (2007). Subcompartments of the macrophage recycling endosome direct the differential secretion of IL-6 and TNFα. *J. Cell Biol.* 178, 57. 10.1083/jcb.200612131 17606866 PMC2064421

[JCS264139C18] Murray, R. Z., Kay, J. G., Sangermani, D. G. and Stow, J. L. (2005). Cell biology: a role for the phagosome in cytokine secretion. *Science* 310, 1492-1495. 10.1126/science.1120225 16282525

[JCS264139C19] Nair-Gupta, P., Baccarini, A., Tung, N., Seyffer, F., Florey, O., Huang, Y., Banerjee, M., Overholtzer, M., Roche, P. A., Tampé, R. et al. (2014). TLR signals induce phagosomal MHC-I delivery from the endosomal recycling compartment to allow cross-presentation. *Cell* 158, 506-521. 10.1016/j.cell.2014.04.054 25083866 PMC4212008

[JCS264139C20] Patente, T. A., Pinho, M. P., Oliveira, A. A., Evangelista, G. C. M., Bergami-Santos, P. C. and Barbuto, J. A. M. (2019). Human dendritic cells: their heterogeneity and clinical application potential in cancer immunotherapy. *Front. Immunol.* 9, 3176. 10.3389/fimmu.2018.03176 30719026 PMC6348254

[JCS264139C21] Pobbati, A. V., Stein, A. and Fasshauer, D. (2006). N- to C-terminal SNARE complex assembly promotes rapid membrane fusion. *Science* 313, 673-676. 10.1126/science.1129486 16888141

[JCS264139C22] Revelo, N. H., Ter Beest, M. and van den Bogaart, G. (2019). Membrane trafficking as an active regulator of constitutively secreted cytokines. *J. Cell Sci.* 133, jcs234781. 10.1242/JCS.234781/224982 31601617

[JCS264139C23] Santalla Méndez, R., Rodgers Furones, A., Classens, R., Fedorova, K., Haverdil, M., Canela Capdevila, M., van Duffelen, A., Spruijt, C. G., Vermeulen, M., ter Beest, M. et al. (2023). Galectin-9 interacts with Vamp-3 to regulate cytokine secretion in dendritic cells. *Cell. Mol. Life Sci.* 80, 306. 10.1007/S00018-023-04954-X 37755527 PMC10533640

[JCS264139C24] Sneeggen, M., Pedersen, N. M., Campsteijn, C., Haugsten, E. M., Stenmark, H. and Schink, K. O. (2019). WDFY2 restrains matrix metalloproteinase secretion and cell invasion by controlling VAMP3-dependent recycling. *Nat. Commun.* 10, 1-20. 10.1038/s41467-019-10794-w 31253801 PMC6599030

[JCS264139C25] Steele, E. M. and Steele, D. S. (2014). Automated detection and analysis of Ca2+ Sparks in x-y image stacks using a thresholding algorithm implemented within the open-source image analysis platform ImageJ. *Biophys. J.* 106, 566-576. 10.1016/j.bpj.2013.12.040 24507597 PMC3944640

[JCS264139C26] Szklarczyk, D., Gable, A. L., Lyon, D., Junge, A., Wyder, S., Huerta-Cepas, J., Simonovic, M., Doncheva, N. T., Morris, J. H., Bork, P. et al. (2019). STRING v11: protein-protein association networks with increased coverage, supporting functional discovery in genome-wide experimental datasets. *Nucleic Acids Res.* 47, D607-D613. 10.1093/nar/gky1131 30476243 PMC6323986

[JCS264139C27] Veale, K. J., Offenhäuser, C., Lei, N., Stanley, A. C., Stow, J. L. and Murray, R. Z. (2011). VAMP3 regulates podosome organisation in macrophages and together with Stx4/SNAP23 mediates adhesion, cell spreading and persistent migration. *Exp. Cell Res.* 317, 1817-1829. 10.1016/j.yexcr.2011.04.016 21586284

[JCS264139C28] Verboogen, D. R. J., ter Beest, M., Honigmann, A. and van den Bogaart, G. (2017). Fluorescence lifetime imaging microscopy reveals rerouting of SNARE trafficking driving dendritic cell activation. *eLife* 6, e23525. 10.7554/ELIFE.23525 28524818 PMC5473687

[JCS264139C29] Verboogen, D. R. J., ter Beest, M., Honigmann, A. and van den Bogaart, G. (2018). Secretory vesicles of immune cells contain only a limited number of interleukin 6 molecules. *FEBS Lett.* 592, 1535-1544. 10.1002/1873-3468.13036 29570778 PMC5969217

[JCS264139C30] Verboogen, D. R. J., Revelo, N. H., ter Beest, M. and Su, B. (2019). Interleukin-6 secretion is limited by self-signaling in endosomes. *J. Mol. Cell Biol.* 11, 144-157. 10.1093/jmcb/mjy038 30016456 PMC6392102

[JCS264139C31] Warner, H., Mahajan, S. and van den Bogaart, G. (2022). Rerouting trafficking circuits through posttranslational SNARE modifications. *J. Cell Sci.* 135, e23525. 10.1242/JCS.260112/276344 35972760

[JCS264139C32] Warner, H., Franciosa, G., van der Borg, G., Coenen, B., Faas, F., Koenig, C., de Boer, R., Classens, R., Maassen, S., Baranov, M. V. et al. (2024). Atypical cofilin signaling drives dendritic cell migration through the extracellular matrix via nuclear deformation. *Cell Rep.* 43, 113866. 10.1016/j.celrep.2024.113866 38416638

[JCS264139C33] Warren, S. C., Margineanu, A., Alibhai, D., Kelly, D. J., Talbot, C., Alexandrov, Y., Munro, I., Katan, M., Dunsby, C. and French, P. M. (2013). Rapid global fitting of large fluorescence lifetime imaging microscopy datasets. PLoS One. 8, e70687. 10.1371/journal.pone.0070687 23940626 PMC3734241

