# Supplementary material for: Phosphorylation of VAMP3 couples IL-6 exocytosis to dendritic cell activation
Source: J Cell Sci. 2025 Oct 14;138(19):jcs264139. doi: 10.1242/jcs.264139 (PMC12579954; doi:10.1242/jcs.264139)
Supplement: Supplementary information [file joces-138-264139-s1.pdf]

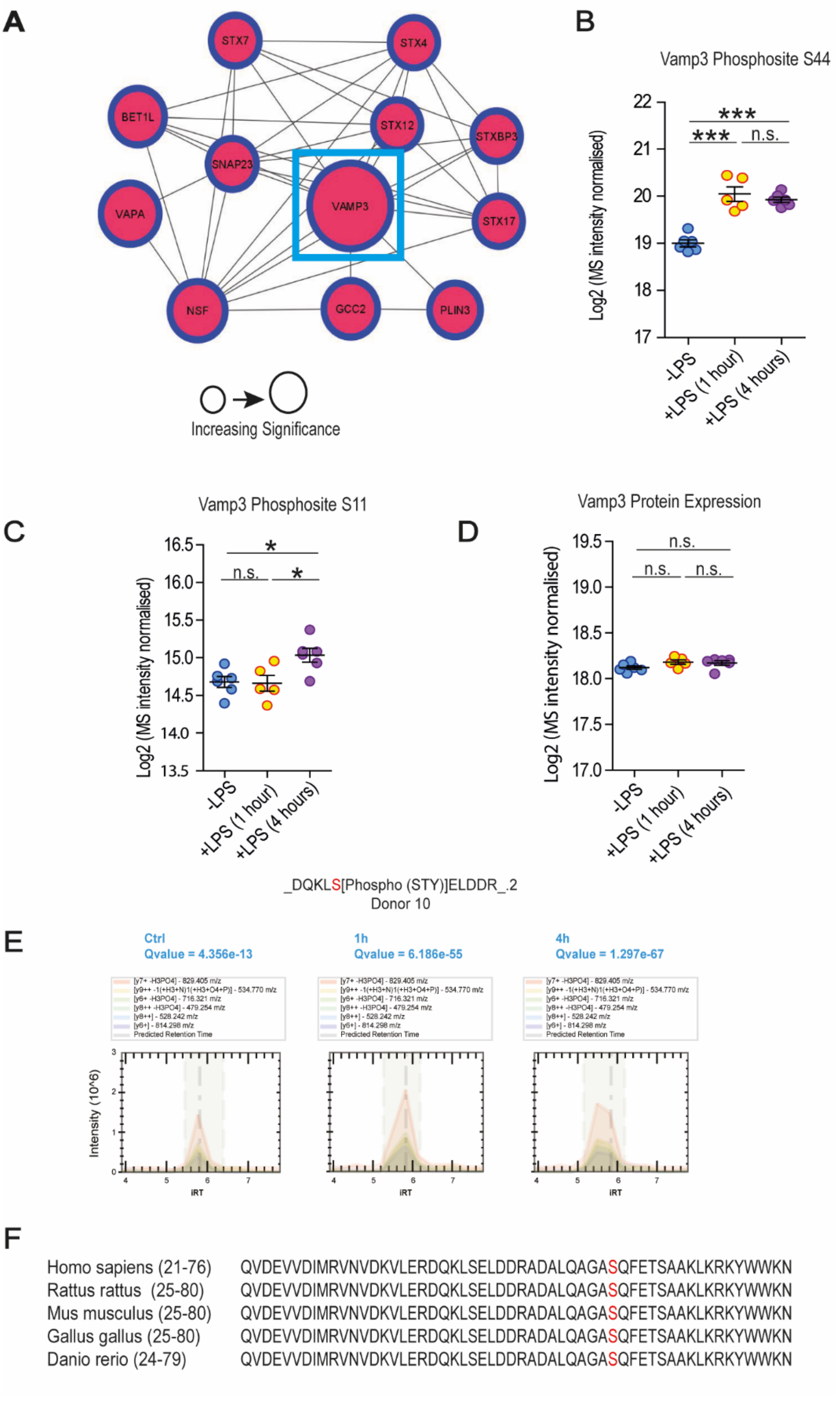

Fig. S1. A Network of SNARE and SNARE-Associated Proteins are Phospho-regulated in Activated Dendritic Cells – data from Warner et al. (2024) for panels A-G. A) Interaction network of SNARE-related proteins found to undergo significant phosphorylation/dephosphorylation events in response to LPS stimulation (at 1 hour and/or 4 hours). Node size indicates relative significance, as calculated by 1/Q value in the original mass spectrometry dataset. Reanalysis of published data (Warner et al. 2024). B) Scatterplot of the normalized MS signal, after Log2 transformation, for phosphorylated serine 44 of VAMP3. C) Scatterplot of the normalized MS signal, after Log2

Journal of Cell Science • Supplementary information

transformation, for phosphorylated serine 11 of VAMP3. D) Scatterplot of the normalized MS signal, after Log2 transformation, for the protein VAMP3. E) Extracted MS2 ion chromatogram for the VAMP3 serine 44 phosphopeptide. Each line represents a fragment ion. iRT = independent retention time (in minutes). F) Alignment of VAMP3 across species reveals a highly conserved region of 55 amino acids, being conserved from human (Homo sapiens) through to mouse (Mus musculus) and even zebrafish (Danio rerio). The phophoserine is marked in red. ANOVA/Tukey multiple comparison tests. *, $P<0.05$; ***, $P < 0.001$; n.s., not significant. For box-and-whisker plots, box represents 25th to 75th percentile, whiskers represent maximum and minimum values, middle band represents data median, and + represents data mean.

Journal of Cell Science • Supplementary information

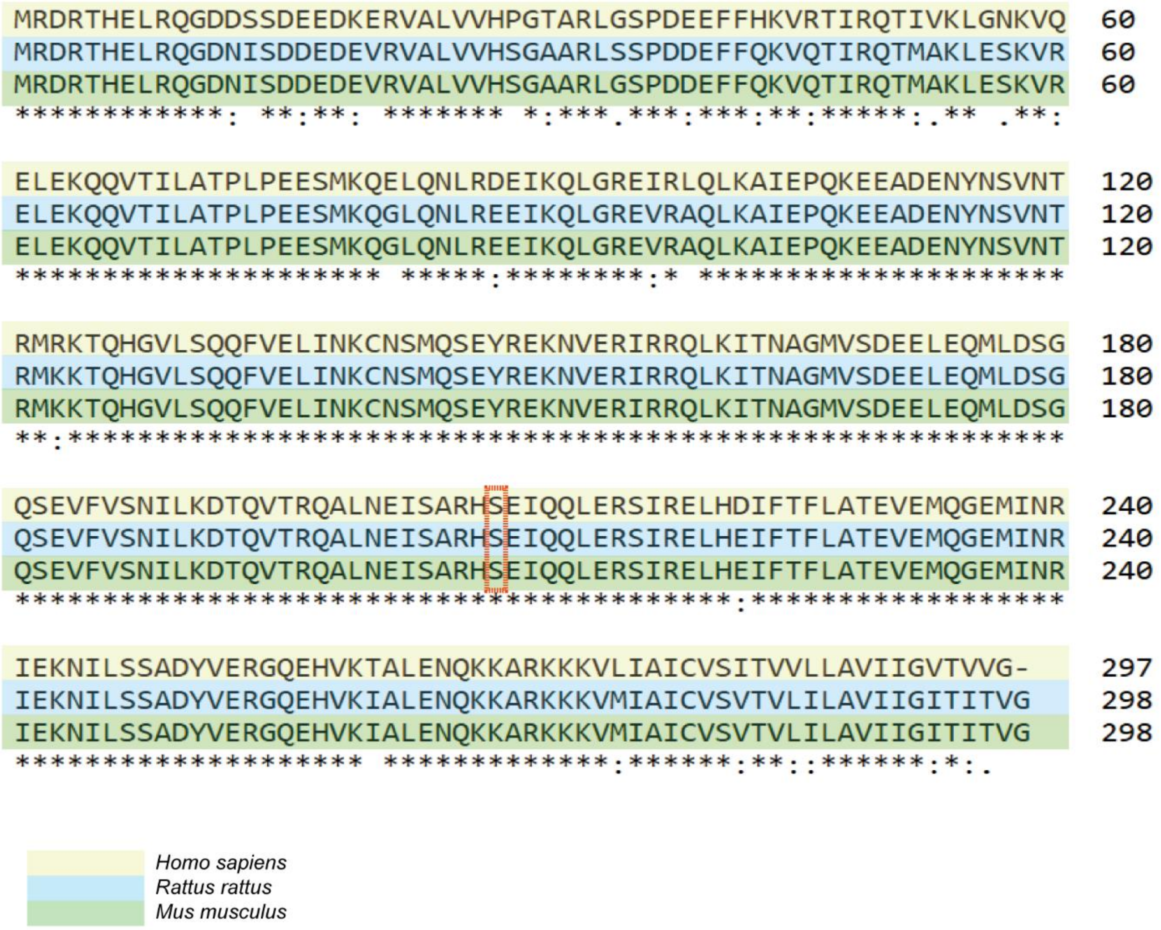

Fig. S2. STX4 is highly conserved. Alignment of human, rat and mouse STX4 amino acid sequences. Human STX4 is 89.56% conserved compared to rat and 89.9% conserved compared to mouse. Serine 208 is highlighted as this was also found to be phosphorylated in response to LPS stimulation (Warner et al. 2024).

Journal of Cell Science • Supplementary information

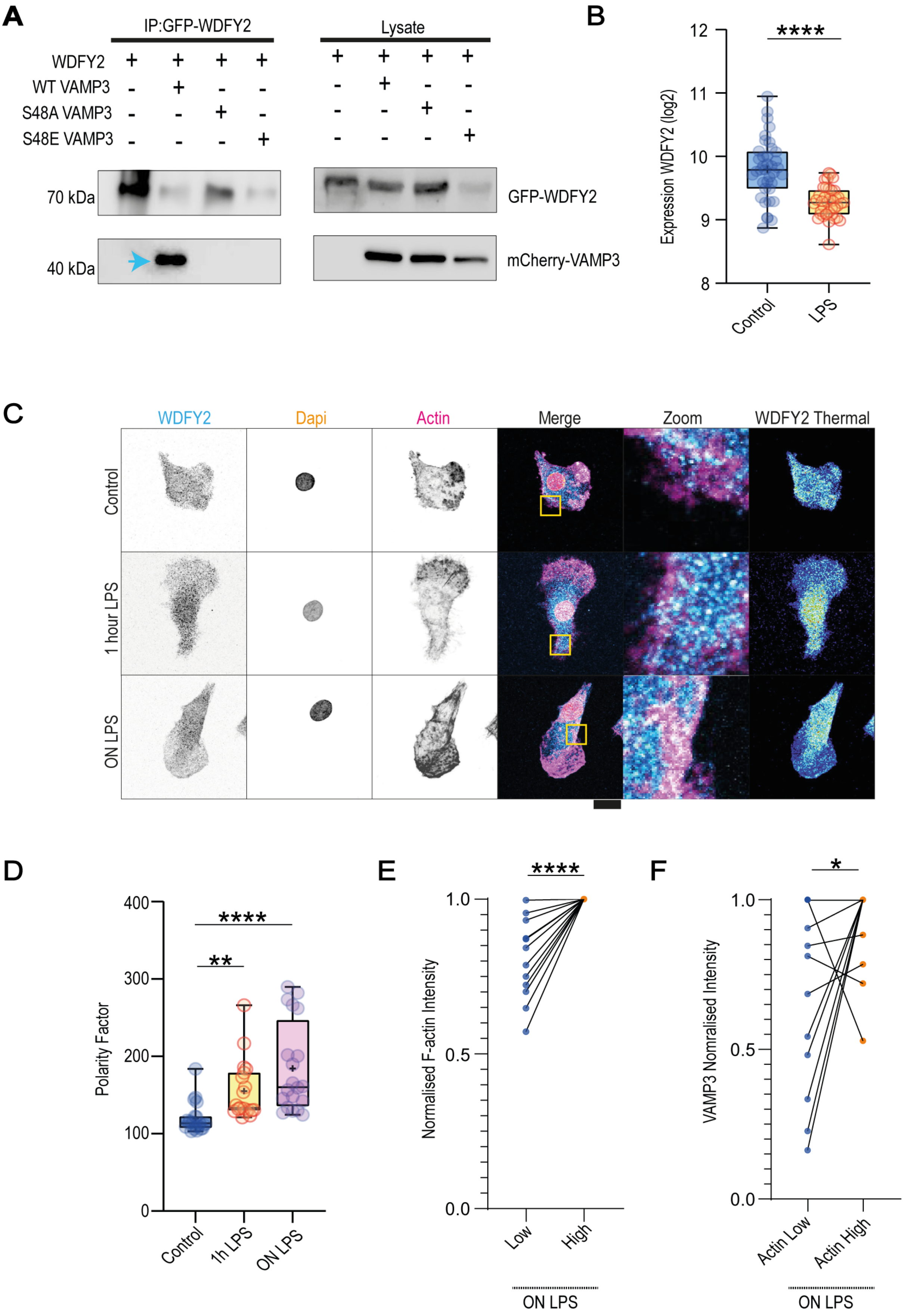
A
IP:GFP-WDFY2
Lysate
WDFY2
WT VAMP3
S48A VAMP3
S48E VAMP3
70 kDa
40 kDa
GFP-WDFY2
mCherry-VAMP3
B
Expression WDFY2 (log2)
****
Control
LPS
C
WDFY2
Dapi
Actin
Merge
Zoom
WDFY2 Thermal
Control
1 hour LPS
ON LPS
D
Polarity Factor
****
**
Control
1h LPS
ON LPS
E
Normalised F-actin Intensity
****
Low
High
ON LPS
F
VAMP3 Nomralised Intensity
*
Actin Low
Actin High
ON LPS

Fig. S3. WDFY2 is LPS sensitive. A) Western blot of immunoprecipitation experiment for dendritic cells expressing WDFY2-GFP with VAMP3-mCherry variants. Immunoprecipitation was performed with an antibody against GFP. Only WT VAMP3-mCherry co-purifies with WDFY2-GFP. B) Expression as measured by microarray genomic profiling (from Lee et al. 2014) of WDFY2 in response to LPS stimulation. Expression shows a small but significant decrease in WDFY2 expression at the mRNA level. C) Example confocal micrograph of dendritic cells immunostained for WDFY2 (cyan) and labelled with phalloidin (magenta) and DAPI (orange). Scale bar indicates 20 µm. D) WDFY2 polarisation increases after 1 hour and overnight LPS stimulation (minimum of 19 measurements condition per over 3 donors) E) F-Actin distribution and F) VAMP3 distribution relative to F-Actin distribution in ON stimulated dendritic cells (12 measurements over 3 donors). Statistical significance calculated using a 2-sided unpaired t-test for panels B, E and F, and ANOVA/Tukey multiple comparison test for panel D. *, $P < 0.05$; **, $P < 0.01$; ****, $P<0.0001$. For box-and-whisker plots, box represents 25th to 75th percentile, whiskers represent maximum and minimum values, middle band represents data median, and + represents data mean.

Journal of Cell Science • Supplementary information

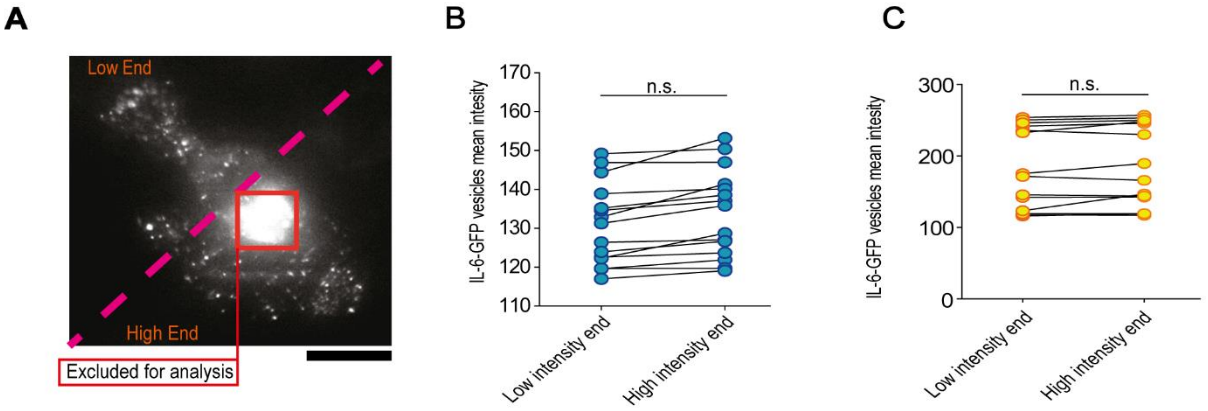

Fig. S4. IL6 Carrying Vesicles are non-polarised in dendritic cells. A) Example TIRF imaging of dendritic cell expressing IL-6-GFP. B) Quantification of IL-6-GFP vesicles at “high” and “low” ends of unstimulated dendritic cells (15 measurements over 3 donors). C) Quantification of IL-6-GFP vesicles at “high” and “low:” ends of dendritic cells stimulated with LPS for ~ 1-4 hours prior to imaging (14 measurements over 3 donors). Scale bar indicates 20 microns. Statistical significance calculated using 2-sided paired t-tests (selected according to the distribution pattern of the data).

Journal of Cell Science • Supplementary information

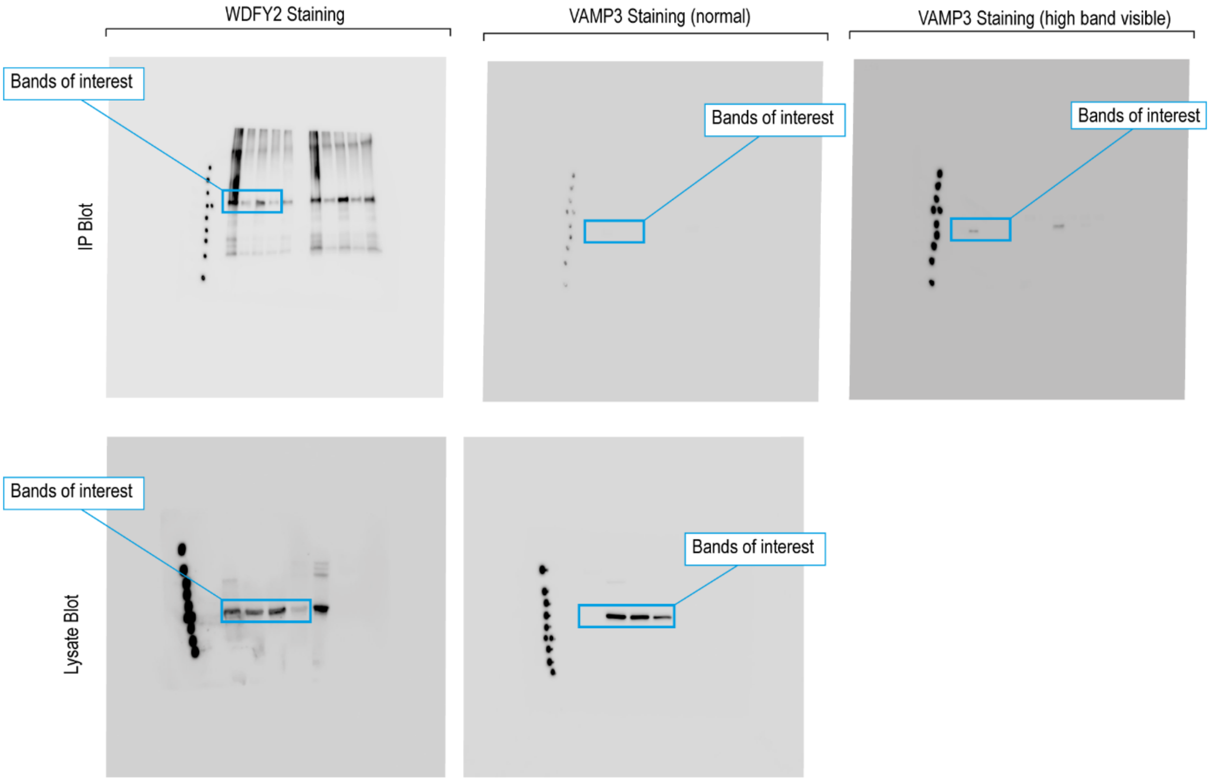

Fig. S5. Blot Transparency – blots from Fig. S3.

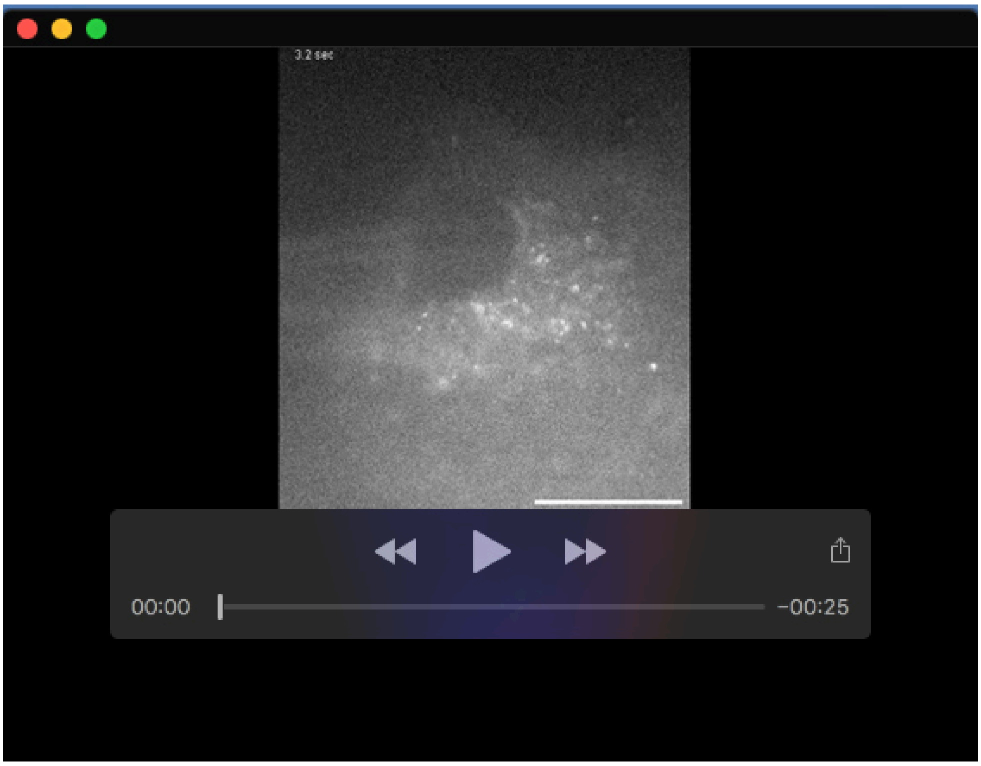

Movie 1. TIRF movie of GFP-IL6 bursts secreted from dendritic cell.

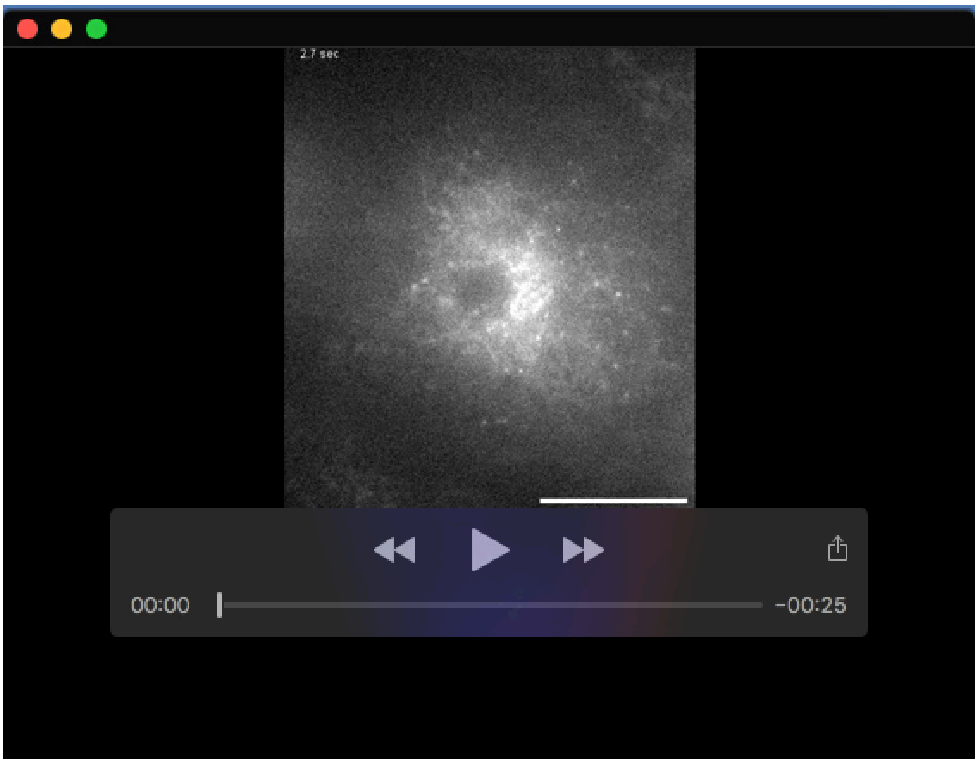

Movie 2. TIRF movie of GFP-IL6 bursts secreted from dendritic cell stimulated briefly with LPS.

Journal of Cell Science • Supplementary information

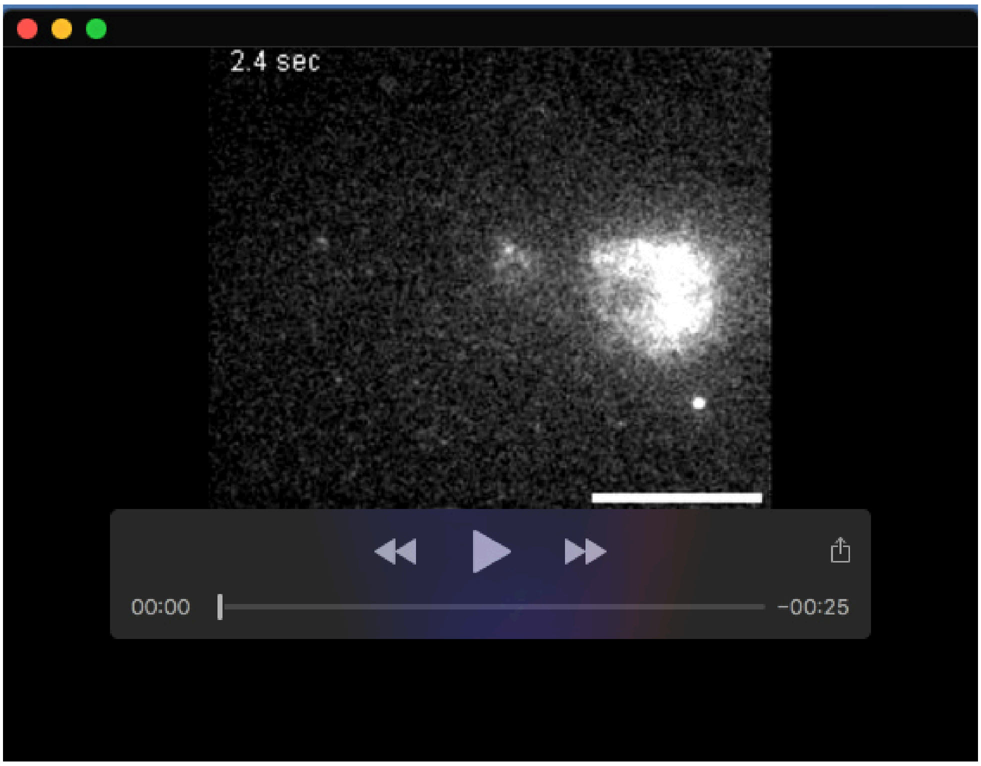

Movie 3. TIRF movie of GFP-IL6 bursts secreted from dendritic cell expressing mCherry-VAMP3 (WT).

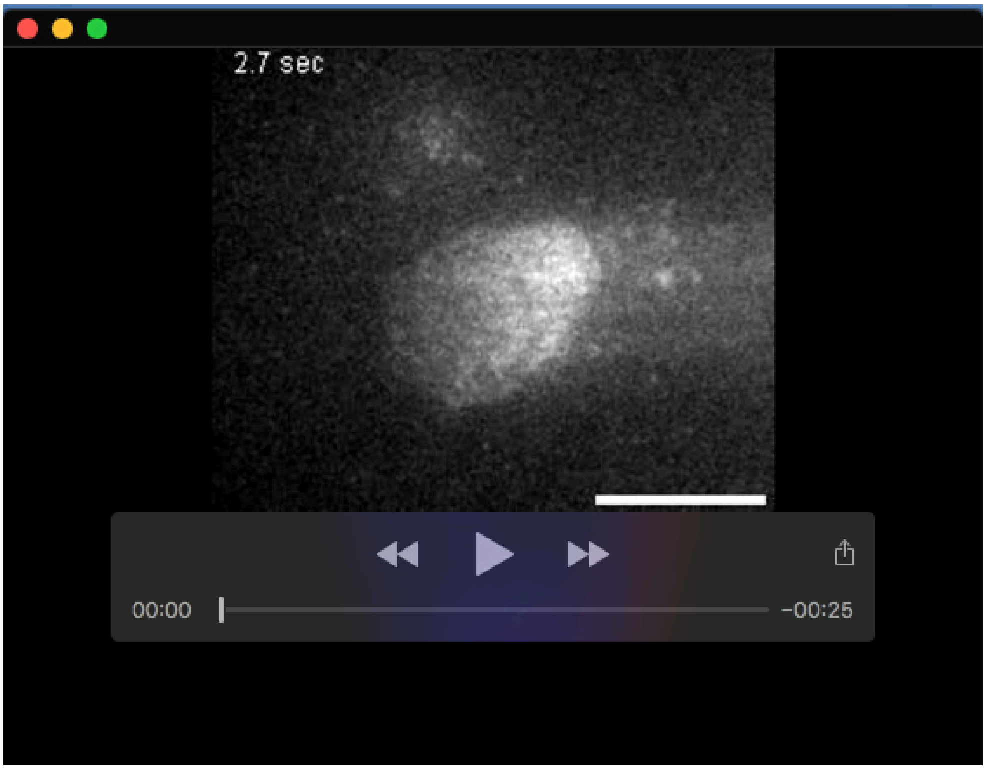

Movie 4. TIRF movie of GFP-IL6 bursts secreted from dendritic cell expressing mCherry-VAMP3 (S48E).
